# Cyclic Fatigue Resistance of Four Heat-Treated Nickel-Titanium Files in Severely Curved Simulated Canals: An In Vitro Study

**DOI:** 10.3390/jcm13195739

**Published:** 2024-09-26

**Authors:** Katia Greco, Gaetano Paolone, Giuseppe Cicero, Giulia Tetè, Nicola Cantile, Maria Teresa Sberna, Teresa Saladino, Enrico Felice Gherlone, Giuseppe Cantatore

**Affiliations:** Department of Dentistry, IRCCS San Raffaele Hospital and Dental School, Vita Salute University, 20158 Milan, Italy; katiagreco@libero.it (K.G.); g.cicero@studenti.unisr.it (G.C.); tete.giulia@hsr.it (G.T.); nicolacantile@yahoo.it (N.C.); sberna.mariateresa@hsr.it (M.T.S.); teresa2108@hotmail.it (T.S.); gherlone.enrico@hsr.it (E.F.G.)

**Keywords:** cyclic fatigue, heat-treated Ni-Ti alloy, martensitic Ni-Ti endodontic instruments, rotary endodontic files

## Abstract

**Background**: Rotary Ni-Ti files are susceptible to sudden intra-canal separation due to cyclic fatigue stress, particularly in curved canals. To increase resistance to cyclic fatigue, new heat-treated files have been introduced. This study aimed to compare the performance of four heat-treated Ni-Ti files in two simulated curved root canals by evaluating the effect of the alloy, rotation speed, and diameter of the files on their resistance to cyclic fatigue. **Methods**: The Ni-Ti files included in the study were the ProTaper Gold^®^ (Dentsply Sirona) F2, ProTaper Ultimate^®^ (Dentsply Sirona) F2, FQ^®^ (Komet) 25.06, and Blueshaper^®^ (Zarc4Endo) Z4 25.06. Two groups of 30 files were selected for each system and were tested in two simulated canals milled in a specific metal template. One group was tested in a 60° curved canal and the other in a 90° curved canal. **Results**: In the 60° simulated canal, there were no instrument fractures within the 15 min time limit. In the 90° simulated canal, the Blueshaper Z4 demonstrated a lower resistance to cyclic fatigue, while FQ 25.06 showed statistically higher fatigue resistance based on both the Kruskal–Wallis and Games–Howell tests (*p* < 0.05). **Conclusions**: No differences were found between files when tested in a 60° curved canal for up to 15 min. However, in a 90° canal, the FQ^®^ files showed significantly higher resistance to cyclic fatigue, especially compared to the Blueshaper^®^ Z4. The ProTaper Ultimate and ProTaper Gold produced intermediate results, with the ProTaper Ultimate F2 slightly outperforming the ProTaper Gold F2.

## 1. Introduction

In endodontic treatments, it is essential to consider the possibility of nickel–titanium (Ni-Ti) files separating within the root canal, as this can result in treatment failure [1]. If a file breaks, it can impede the thorough shaping and disinfection of the root canal system, leaving some areas untreated and contaminated [2]. The likelihood of separating Ni-Ti files is significantly higher for rotary files than manual files due to the development of torsional and fatigue stresses [2]. To reduce the risk of torsional failure, preliminary preparation is essential, such as establishing a glide path in the root canal and carefully using shaping files [1]. However, fatigue stress can occur suddenly, even with new files. Cyclic fatigue failures mainly occur due to the continuous rotation of Ni-Ti files in curved canals or in the presence of severe coronal interference [3]. Indeed, when mechanically rotated in curved canals, Ni-Ti files undergo repeated cycles of tensile and compressive stress, resulting in micro-cracks on the surface. Over time, these cracks can deepen, leading to sudden file separation [3,4]. The severity of cyclic fatigue stress depends on several factors, such as the root canal characteristics (curvature angle, curvature radius, presence of a glide path), motor rotation speed and torque [4,5,6], and the geometric design of the file [7,8]. Over the years, manufacturers have focused on developing ideal instruments with excellent resistance to flexural stresses. Initially, attention was focused on the geometric characteristics of files, such as the taper, diameter, cross-sectional design, blade design, pitch, and helix angle. Modifying some of these factors (e.g., the central core, pitch, and helix angle) made it possible to significantly improve the fatigue resistance of rotary files [9,10]. In recent years, research has been focused on creating files using higher-performing Ni-Ti alloys that undergo thermal/thermomechanical treatments. These treatments aim to enhance the flexibility and resistance of the files by adding a layer of titanium dioxide, which can change the file’s color [11,12]. One of the main advantages of these thermo-mechanical treatments is that they alter the phase-transformation temperatures of the Ni-Ti alloy, thereby improving specific properties at the temperatures commonly encountered in clinical use [13,14]. The distinct color of the alloy indicates specific thermal treatments, each of which results in targeted modifications in the instrument’s properties. For example, the blue color treatment enhances the flexibility of the file, improving its performance in shaping curved root canals. On the other hand, the gold color treatment optimizes its resistance to cyclic fatigue, which is crucial in preventing instrument fracture during prolonged use. Additionally, the pink-colored alloy undergoes a treatment to enhance its strength and durability during the initial negotiation and shaping of the root canal, especially in challenging anatomical conditions [14]. Several studies have shown that thermal treatments can significantly improve the cyclic fatigue resistance of Ni-Ti instruments. Research by Pereira et al. [14,15], Karataşlıoğlu E. et al. [16], and Zinelis et al. [17] has demonstrated that thermally treated files, similar to the ones examined in our study, exhibit better resistance to cyclic fatigue than conventional Ni-Ti files. Grande et al. [18] confirmed that thermally treated files exhibit greater cyclic fatigue resistance than non-thermally treated files. Tabassum et al. [19] emphasized that the thermal treatment of Ni-Ti alloys signifies a noteworthy advancement in endodontic instrument design, providing clinical benefits such as increased fracture resistance and extended instrument life. In our study, we evaluated the resistance to cyclic fatigue of four heat-treated Ni-Ti files in simulated curved root canals with different angles and radii of curvature. These files have a tip diameter of 0.25 mm and tapers ranging from 0.06 to 0.08. Our research is unique because we compared the cyclic fatigue resistance of four thermally treated Ni-Ti instruments with different tapers in simulated curved canals with varying angles and radii. We also tested the FQ file, a recently introduced product with limited existing research, such as the Blueshaper Z4 instrument. Additionally, we included the ProTaper Ultimate files in our study to determine if the changes from the older ProTaper Gold led to increased stress resistance. We specifically compared files with a 6% taper at the tip (FQ files) to files with an 8% taper (ProTaper Ultimate and ProTaper Gold) to examine the effect of the taper in the apical 3 mm of the files on cyclic fatigue strength. This is in contrast to most studies that have focused on instruments of the previous generation, often considering only individual file systems or root canal curvatures but rarely comparing resistance to fatigue stresses in two different canal curvatures and the effects of the taper of the apical part of the file on the resistance to fatigue stresses. The aim of this study is to compare the resistance to cyclic fatigue stresses of four different nickel–titanium files. Although these instruments have the same tip diameters, they vary in alloy type, taper, and blade design. The null hypothesis of this study posits that there are no statistically significant differences in the cyclic fatigue strength of the tested instruments.

## 2. Materials and Methods

We utilized four different systems of rotary Ni-Ti files connected to an X-Smart Plus endodontic motor (Dentsply Sirona). The files were operated at the recommended rotation speed and torque (N·cm) as specified by the manufacturers.

(1) ProTaper Gold^®^ F2 (Dentsply Sirona, Charlotte, NC, USA): This file is made of heat-treated gold Ni-Ti alloy. It has a convex triangle cross-sectional design, a tip diameter of 0.25 mm, a regressive taper (8% for the apical 3 mm), and a maximum diameter of 1.05 mm. The ProTaper Gold F2 was used at 300 rpm with the motor torque set at 3.00 N·cm.
(2) ProTaper Ultimate^®^ F2 (Dentsply Sirona, Charlotte, NC, USA): This file is made of heat-treated gold Ni-Ti alloy. It has a parallelogram-shaped cross-sectional design with a variable cutting angle, a tip diameter of 0.25 mm, a regressive taper (8% for the apical 3 mm), and a maximum diameter of 1.00 mm. The ProTaper Ultimate F2 was used at 400 rpm with the motor torque set at 4 N·cm.
(3) File FQ^®^) 25.06 (Komet Dental, Rock Hill, SC, USA): This file is made of heat-treated alloy and has a double-S variable cross-section, a non-cutting tip, a tip diameter of 0.25 mm, and a maximum diameter of 0.90 mm with a fixed taper of 6%. The FQ^®^ was used at 300 rpm with the motor torque set at 2.4 N·cm.
(4) Blueshaper^®^ (Zarc4Endo, Asturias, Spain) Z4 25/06: This file is made of heat-treated blue Ni-Ti alloy and has a convex triangle cross-sectional design, a partially cutting tip, a tip diameter of 0.25 mm, a maximum diameter of 0.90 mm, and a taper of 6%. The Z4 was used at 500 rpm with the motor torque set at 4 N·cm.

Two groups of 30 files were selected for each instrument system. One group was tested in simulated canals with a curvature angle of 60°, while the other was tested in canals with a curvature angle of 90°. The decision to include 30 samples per group was based on the need to improve the statistical strength of the analysis and minimize the margin of error. Scientific research indicates that increasing the sample size can greatly enhance the accuracy of the results when studying the cyclic fatigue resistance of endodontic instruments. In particular, larger sample sizes increase the statistical power, making it possible to detect even subtle differences [20,21]. To determine the required sample size, we examined various studies on the cyclic fatigue resistance of Ni-Ti instruments. Previous research has employed sample sizes ranging from 20 to 30 samples per group to ensure statistically robust and reliable results [9,22,23,24,25,26,27,28,29,30,31]. To conduct an initial assessment, we utilized G*Power software version 3.1.9.7, a widely used tool for calculating the necessary sample size based on statistical power, significance level, and expected effect size. With a sample size of 30 units per group, we estimated that we could detect significant differences with a statistical power greater than 80%, a significance level (α) of 0.05, and a moderate effect size (f = 0.25). This evaluation confirmed that a sample size of 30 units per group was adequate to meet our research objectives and provide reliable results.

**Figure 1:**
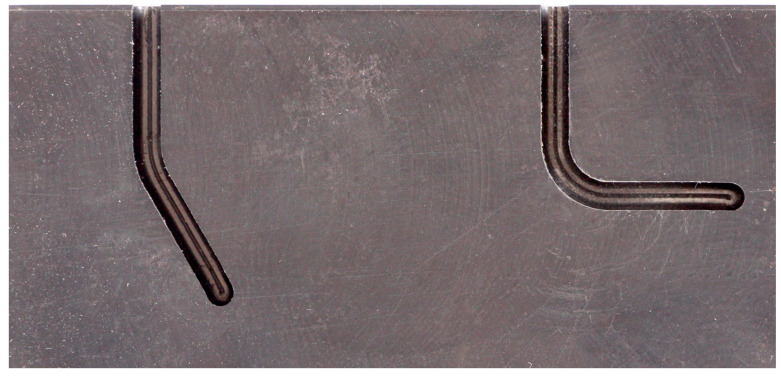
The metal block with the two milled canals.

A metal template was made to test the files’ resistance to cyclic fatigue without interfering with the canal walls during rotation. This template featured two canals milled on its surface. The methods used to prepare the metal template and assess the files’ resistance were previously described in studies by Gambarini et al. [22], Al-Sudani et al. [32], and Whipple et al. [33]. To make the template, a 60 mm high, 40 mm wide, and 20 mm deep metal block made of X155 steel was constructed using computer-aided design in collaboration with a precision micro-milling center. Two curved canals were milled in the metal block, as shown in Figure 1. The canals have the following characteristics: Canal 1: curvature angle, 60°; curvature radius, 5 mm; canal length, 25 mm; canal diameter, 2 mm; milling depth, 3 mm; and Canal 2: curvature angle, 90°; curvature radius, 3 mm; canal length, 25 mm; canal diameter, 2 mm; milling depth, 3 mm.

**Figure 2:**
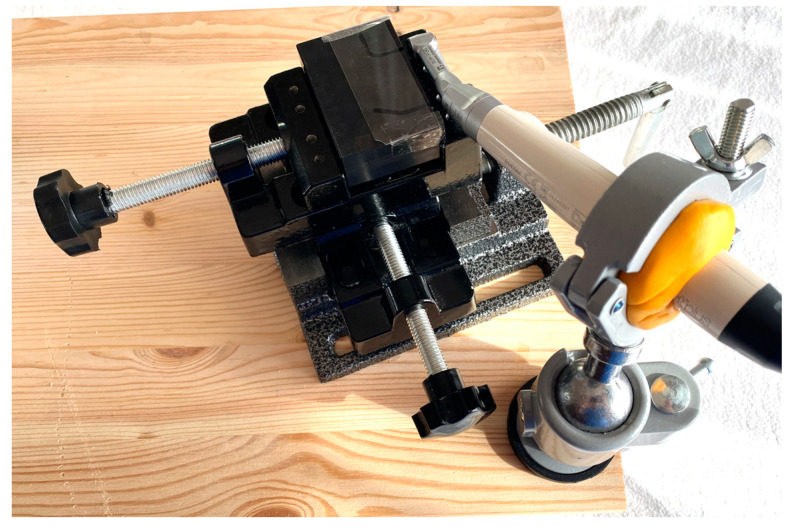
Fastening system for the metal block.

The steel template was milled to an accuracy of ± 0.001 mm and then tempered at a temperature above 1000 °C to achieve a hardness index of 61 ± 2 HRC. X155 steel is a high-performance, wear-resistant material widely used to produce industrial machine-cutting tools. The hardening process further increases the wear resistance. The material used to create the template was not affected by the continuous rotation of the Ni-Ti files, thus preserving the original geometry of the root canals. The endodontic motor was secured to the metal template using bench vises to ensure reliable results, allowing transverse movement. The bench vises were attached to a wooden table, and the endodontic motor was affixed to the bench using a vise equipped with a ball connector (Figure 2).

Each instrument was fully inserted into simulated canals and activated. A maximum testing time of 15 min was set. The movement of each file was recorded with a video camera until a fracture occurred or until the time limit was reached if there was no fracture. Using video-editing software (iMovie 10.4.2; Apple Inc., Cupertino, CA, USA), we could accurately determine the time to fracture for each file. The number of cycles to fracture (NCF) was calculated using the formula NCF = time to fracture (s) × RPM/60. Statistical analysis was conducted using IBM^®^ SPSS^®^ Statistics software version 28 (IBM Corp., Armonk, NY, USA). The average values and standard deviation for each family of instruments were calculated, and Levene’s test was used to assess the equality of variances among the groups. As the assumption of homogeneity of variances was violated, the non-parametric Kruskal–Wallis test was conducted to determine whether all samples originated from the same distribution. Subsequently, the Games–Howell test was used to compare all possible pairwise combinations of group differences, as it can handle unequal variances and varying sample sizes [20,21].

## 3. Results

No fractures occurred within the 15 min time limit in the 60° simulated canal. However, notably different results were observed in the 90° curved canal. Table 1 presents the average time to fracture in seconds and minutes and the average number of cycles to failure (NCF) for the tested instruments.

Levene’s test was conducted to assess whether the variances among the groups were equal. The F-value is used for this assessment, and a higher F-value indicates more significant differences in variance. The *p*-value shows the likelihood of equal variances, and a *p*-value less than 0.05 indicates a significant difference, justifying the non-parametric methods. The null hypothesis of Levene’s test is that the variances across the groups are equal (homogeneous). The results rejected this hypothesis (F = 9.856, *p* < 0.05), indicating the need for non-parametric methods for further analysis. Table 2 summarizes each group’s average, minimum, and maximum NCF values and the standard deviation. Additionally, it includes the results of Levene’s test for equality of variances (F = 9.856, *p* < 0.05).

The Kruskal–Wallis test was used to assess the differences between groups. This test checks if all group distributions are equal. The H-statistic is the test statistic used to determine if there are significant differences in cyclic fatigue resistance (NCF) among the groups. Degrees of Freedom (df) refers to the number of groups minus one. A *p*-value < 0.05 indicates that at least one group differs significantly. The test results showed a highly significant difference among the groups (H = 83.53, *p* < 0.001), as detailed in Table 3 indicating that we can reject the null hypothesis. This means that at least one group differs significantly from the others in terms of cyclic fatigue resistance.

After that, the Games–Howell test was used to compare all possible pairwise combinations of group differences, as explained in previous studies [23,24]. This test examines each pair of groups to determine if there are statistically significant differences in fracture times and revolutions to failure (NCF). In this case, the null hypothesis is that there are no differences between the means of the paired groups. The Games–Howell test indicated that all pairwise comparisons revealed statistically significant differences in cyclic fatigue resistance (*p* < 0.05), with 95% confidence intervals for the differences not including zero (Table 4). Therefore, we can reject the null hypothesis for all pairwise group comparisons, confirming the significant differences observed.

## 4. Discussion

Despite significant advancements in endodontic instruments, cyclic fatigue stress remains a major cause of unexpected intra-canal instrument separation. This issue can significantly impact treatment outcomes, prompting manufacturers to focus on redesigning instruments and finding better alloys. Through thermal and thermomechanical treatments, more flexible Ni-Ti alloys have been developed, which reduce shape memory and enhance fatigue resistance [10,12,14,15,16,17,23,25,26,29,31,34,35,36,37]. This study compared the performance of four different heat-treated Ni-Ti instrument systems when used in curved canals. Two groups of 30 instruments were chosen for each system, as described in the Materials and Methods Section. For all files, the tip diameter was 0.25 mm. This decision was based on years of clinical experience and existing literature. It is worth noting that most studies evaluating cyclic fatigue use files with a 0.25 mm tip diameter [23,24,27,28,29,30,34,37,38]. Using smaller instruments (0.20 mm) may lead to inadequate cleaning of the apical area and insufficient irrigant space.

Conversely, files with a tip diameter of 0.30 mm are generally not recommended for severe curvatures due to their increased stiffness. For each file, a maximum testing time of 15 min was established. Setting this time limit is based on previous studies in the field, which have shown that most fractures due to continuous use occur within a few minutes, especially under severe canal curvature conditions. For example, Lopes et al. [39] observed that Ni-Ti instruments tend to break within the first 10 min of use in extreme conditions. Similarly, Ubaed et al. [25] found that testing for longer than 10–15 min does not provide significant additional information. They noted that instruments exceeding this time frame typically exhibit very high resistance, which is not likely to be replicated in routine clinical scenarios. Therefore, the 15 min mark was chosen because it is long enough to detect most fractures while minimizing issues such as overheating or excessive wear. Furthermore, the time limit of 15 min was chosen to replicate real-life usage conditions for Ni-Ti instruments in endodontic procedures. In routine clinical practice, these instruments are typically not used for extended periods in a single canal, particularly in critical areas of the root canal system. Prolonged usage of an instrument in a single canal for more than a few minutes is discouraged to minimize the risk of breakage and maintain effectiveness. Standardizing a 15 min testing period makes comparing instruments with varying resistance characteristics and designs across different study groups feasible. The instruments were tested in simulated canals with a 2 mm diameter to minimize torsional stress and focus solely on cyclic fatigue resistance. Gambarini et al. [22] and Rubio et al. [23,24] used a metal block with similar characteristics. All the files in the study presented a tip diameter of 0.25 mm and an apical taper between 6% (FQ files) and 8% (ProTaper Ultimate). In the 60° curvature tests, none of the instruments fractured within the 15 min time limit, indicating strong resistance under moderate stress conditions. However, when tested in a more severe 90° curvature, significant differences in performance emerged. The Kruskal–Wallis test for differences in the NCF revealed a highly significant difference among the groups. The Games–Howell test indicated that all pairwise comparisons between instruments revealed statistically significant differences in cyclic fatigue resistance (*p* < 0.05). If we examine the results for the Blueshaper Z4, we find that the files performed less effectively than any other systems in the 90° curved canals. Indeed, the Blueshaper Z4 exhibited a significantly lower cyclic fatigue resistance with an average number of cycles to fracture (NCF) of 20.467 vs. a mean NCF of 37.97 for ProTaper Gold, NCF of 45.10 for ProTaper Ultimate, and 55.73 for FQ files (see Table 2). In contrast to our findings, Rubio et al. [23] found that the Blueshaper Z4 exhibited high resistance to cyclic fatigue. However, they report a convex triangle cross-section design for the ProTaper Ultimate [23]. This information is incorrect since the ProTaper Ultimate presents a parallelogram-shaped cross-section. Furthermore, in their study, the authors used the Z4 at a speed of 350 rpm and only in canals with a 60° curvature angle. Our study followed the manufacturer’s recommendations and used the Z4 at a rotation speed of 500 rpm in canals with 60° and 90° curvature angles. In summary, the better results obtained by Rubio et al. with the Z4 [23] may depend on the lower rotation speed and their use in a canal with a less-severe curvature. Gao Y et al. [26] confirmed that thermally treated alloys, such as those used in the Blueshaper Z4, can experience changes in fatigue resistance depending on the rotation speed and canal curvature. This suggests that using the Ni-Ti files at lower rotation speeds might enhance fatigue resistance, as confirmed by Lopes et al. [34,38], MuKhlif [40], and Faus-Matoes et al. [27]. Furthermore, the diameter and blade design of the files could explain the discrepancies among apparently similar studies [41]. Over 20 years ago, Zelada et al. [5] demonstrated that fatigue stress depends not only on the angle and radius of the curvature of the root canal but also on the rotation speed, diameter, taper, and cross-sectional design of the file. Castelló-Escrivá et al. [28] studied the innovations in Ni-Ti rotary file systems and found that the design and thermal treatment of the core has a significant impact on the clinical performance of these instruments. In a separate study, Di Nardo et al. [29] compared the fatigue resistance of two nickel–titanium rotary instruments, AF Blue S4 and S-One, from Fanta Dental CO., Ltd., Shanghai, China. These instruments underwent the same heat treatment and grinding procedure but had different cross-section designs. AF Blue S4 has a convex triangular cross-section with three cutting blades, while S-One has an S-shaped cross-section with two cutting blades. The instruments tested showed a tip diameter of 0.25 mm and a fixed 6% taper and were tested in a 90° curvature artificial canal. In other words, they were identical except for the cross-section design. The results demonstrated a significantly higher resistance for the S-One file with an S-shaped cross-section. The study by Ersoy et al. on Ni-Ti files with a 4% fixed taper reported similar results, where instruments with a double S cross-section had the highest resistance to cyclic fatigue among the groups [35]. The Blueshaper files have a convex triangle cross-section (similar to the AF Blue S4), which may explain their lower resistance to fatigue in 90° curved canals along with the higher rotation speed. The FQ 25.06 files, with a cyclic fatigue resistance of 54.71 ± 8.42, outperformed the other three systems tested. They have an S-shaped cross-section, which could be one of the reasons for their higher fatigue resistance. This result is consistent with the findings from Generali et al. [30], who highlighted that thermally treated Procodile Q files (Komet Dental, Rock Hill, SC, USA), similar to the FQ files, exhibit superior cyclic fatigue resistance due to increased flexibility and reduced shape memory. Unfortunately, there is a lack of investigations on FQ files in the literature, so no other information is available on their performance in curved canals. In summary, the reasons for the better results of the FQ files are their lower rotation speed, an S-shaped cross-section, and the alloy “Q Ni-Ti heat-treated alloy”, whose features are not declared by the manufacturers. This study found similar results between the ProTaper Ultimate F2 (mean NCF 45.10) and the ProTaper Gold F2 (mean NCF 37.97), suggesting that these instruments offer comparable cyclic fatigue resistance. However, the Games–Howell test for pairwise comparisons revealed significant differences between the two files (*p*-value < 0.05). This finding aligns with Martins et al. [36], who recently compared the ProTaper Ultimate and ProTaper Gold and found that PT Ultimate instruments had lower torsional strength but greater flexibility than the ProTaper Gold instruments. Additionally, they observed that as the sizes of the instruments increased, there was a progressive increase in the maximum torque, rotation angle, and bending loads. In a similar study by Diaconu et al. [42], ProTaper Ultimate files performed better than ProTaper Gold files regarding the times to failure and the number of cycles to failure (NCF) using continuous rotation and reciprocating motions. Finally, the study focused on the taper of the file. Four files with a diameter of 0.25 mm in D_1_ were compared. The files had different tapers: a fixed 6% taper for the FQ file, a taper ranging from 6% to 5% for the Blueshaper Z4, and a taper ranging from 8% in D1 down to 3% in D16 for the ProTaper Gold F2 and ProTaper Ultimate F2. The taper affects the file’s stiffness, bending, and torsional behavior. A higher taper increases the torsional rigidity but reduces the bending flexibility and resistance to fatigue stresses [31,37,43]. The importance of taper for flexibility raises a question: why was the Blueshaper Z4 (taper 5–6%) less resistant to cyclic fatigue than the ProTaper Gold F2 and Ultimate F2 (taper 8%)? The explanation is that multiple causes of fatigue failures play different roles in clinical situations. When dealing with severely curved canals, it is important to use new instruments, choose a file system with a maximum taper of 4–5%, and reduce the rotation speed and torque of the motor up to a maximum of 250–300 rpm. Last-generation motors with “optimum torque reverse” can reduce the file load. Considering the study’s limitations, it is important to note that it only evaluated rotary files, not reciprocating files. Additionally, it only included files with a diameter of 0.25 mm in D1. Furthermore, the study only examined heat-treated martensitic files and did not compare them with austenitic files manufactured using different alloys (such as M-Wire). The study also did not account for the effect of coronal interferences on the files’ cyclic fatigue resistance.

As a result, potential areas for further investigation should include the following:

- Comparing the fatigue stress resistance of rotary and reciprocating new-generation files—Comparing the fatigue stress resistance of martensitic (heat-treated) and austenitic Ni-Ti files;
- Creating simulated canals with coronal interferences to evaluate their effect on fatigue stress;
- Replicating this study using the same files but with different tip diameters (0.20 mm and 0.30 mm).

## 5. Conclusions

The study found that subjecting Ni-Ti files to heat treatment increases their resistance to cyclic fatigue. When tested in a 60° curved canal for up to 15 min, no files separated, and no differences were observed between the files. However, in a 90° canal, significant differences were found among all groups. The FQ files showed significantly higher resistance to cyclic fatigue than the other groups, especially when compared to the Blueshaper^®^ Z4. The results of the ProTaper Ultimate and ProTaper Gold were similar, but statistical analysis revealed that the ProTaper Ultimate F2 was significantly more resistant than the ProTaper Gold F2. Therefore, the study’s null hypothesis (no significant difference in the cyclic fatigue resistance between the different heat-treated files) can be rejected.

## Figures and Tables

**Table 1:** Average times to failure (in seconds and minutes) and average revolutions to failure for the instruments tested.

Instrument	Time to Failure (Seconds)	Time to Failure (Minutes)	Revolutions to Failure (NCF)
**Blueshaper Z4**	2.46	0.04	20.46
**ProTaper Gold F2**	7.60	0.13	37.97
**ProTaper Ultimate F2**	6.80	0.11	45.10
**FQ 25.06**	8.16	0.14	55.73

**Table 2:** Average, minimum, and maximum NCF (Revolution to failure) values, the standard deviation for each group, along with the results of Levene’s Test for Equality of Variances (F = 9.856, *p* < 0.05). This indicates the need for non-parametric methods for further analysis.

Parameters	Blueshaper Z4	ProTaper Gold F2	ProTaper Ultimate F2	FQ 25.06
**Mean NCF**	20.46	37.97	45.10	55.73
**Standard Deviation NCF**	1.73	9.02	4.43	6.31
**Minimum NCF**	17.83	36.75	38.53	48.91
**Maximum NCF**	22.58	40.20	48.91	68.58
**Levene’s test F-value and *p*-value**	F= 9.856, *p*-value < 0.05			

**Table 3:** Kruskal–Wallis test for differences in NCF. The test revealed a significant difference among the groups (H = 83.53, *p* < 0.001).

Test Statistic	Degrees of Freedom (df)	*p*-Value
H-statistic. 85.83	3	<0.001

**Table 4:** Games–Howell post hoc test for pairwise comparisons. The results confirm the significance of the differences between each pairwise group.

Groups	Mean Difference	Confidence Intervals 95%	*p*-Value	Rejection of the Null Hypothesis
Blueshaper vs. ProTaper Gold	−5.1513	−6.23, −4.07	<0.05	True
Blueshaper vs. ProTaper Ultimate	−4.1023	−5.18, −3.02	<0.05	True
Blueshaper vs. File FQ	−3.3657	−4.44, −2.28	<0.05	True
ProTaper Gold vs. ProTaper Ultimate	1.0490	5.78, 1.52	<0.05	True
ProTaper Gold vs. File FQ	1.7857	1.31, 2.25	<0.05	True
ProTaper Ultimate vs. File FQ	0.7367	0.26, 1.21	<0.05	True

## Data Availability

No new data were created or analyzed in this study. Data sharing is not applicable to this article.
